# Supplementary material for: Complex‐centric proteome profiling by SEC‐SWATH‐MS
Source: Mol Syst Biol. 2019 Jan 14;15(1):e8438. doi: 10.15252/msb.20188438 (PMC6346213; doi:10.15252/msb.20188438)
Supplement: Supplementary file 8 — Dataset EV7 [file MSB-15-e8438-s008.zip › feature_plots_string/Q9Y5P8.pdf]

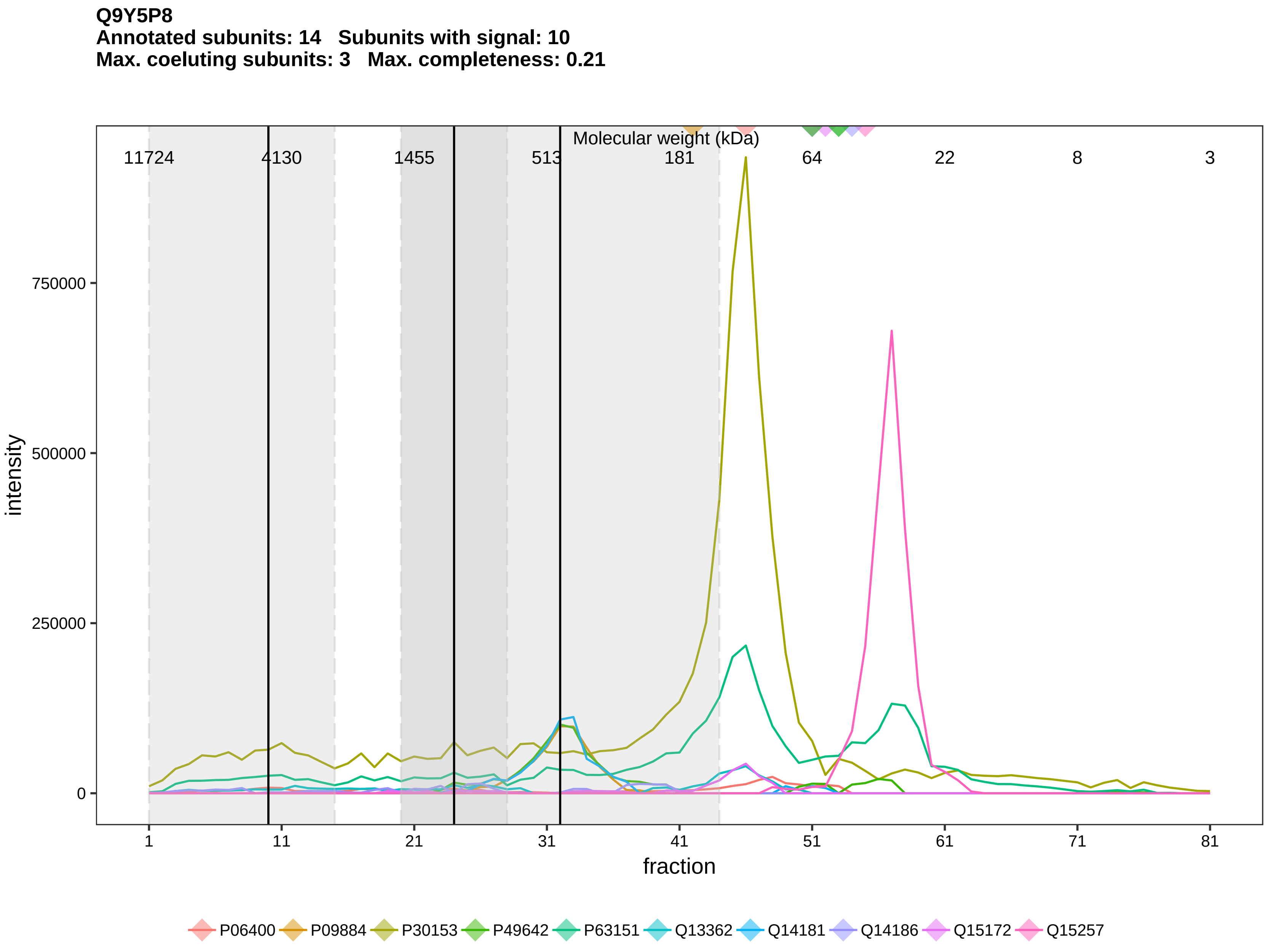
Q9Y5P8
Annotated subunits: 14 Subunits with signal: 10
Max. coeluting subunits: 3 Max. completeness: 0.21
Molecular weight (kDa)
11724
4130
1455
513
181
64
22
8
3
intensity
750000
500000
250000
0
fraction
1
11
21
31
41
51
61
71
81
P06400
P09884
P30153
P49642
P63151
Q13362
Q14181
Q14186
Q15172
Q15257
